# Greater Positivity Resonance Is Associated With Better Mental Health in Dementia Caregivers

**DOI:** 10.1093/geroni/igaf122.290

**Published:** 2025-12-31

**Authors:** Jenna Wells, Suzanne Shdo, Claire Yee, Diana Heath, Barbara Fredrickson, Robert Levenson

**Affiliations:** Cornell University, Ithaca, New York, United States; University of California, Berkeley, Berkeley, California, United States; Mayo Clinic, Scottsdale, Arizona, United States; Arizona State University, Tempe, Arizona, United States; The University of North Carolina at Chapel Hill, Chapel Hill, North Carolina, United States; University of California, Berkeley, Berkeley, California, United States

## Abstract

As a group, family caregivers of people with dementia (PWDs) have substantially elevated levels of depression and anxiety, compared to same-aged non-caregiving adults. However, there is striking variation in mental health among individual caregivers. Previous research on predictors of the sources of these individual differences has focused on characteristics of individual caregivers and PWDs (rather than on the caregiver-PWD relationship), self-report measures (rather than behavioral measures), and negative emotion (rather than positive emotion). We examined 185 caregiver-PWD dyads who had an unrehearsed 10-minute conversation about an area of conflict in the laboratory. Video recordings were coded for Behavioral Indicators of Positivity Resonance (BIPR), which derives from Positivity Resonance Theory and assesses dyad-level behavioral expressions of positive affect, mutual warmth, and synchrony. Caregivers reported their level of depression, anxiety, and emotional well-being and PWDs underwent a comprehensive neurological and neuropsychological evaluation. Pearson’s correlations revealed that greater dyad-level BIPR was associated with caregivers’ higher emotional well-being (r(181) = .19, p = .008), lower depression (r(179) = -.17, p = .025), and lower anxiety (r(180) = -.15, p = .045). Regression analyses revealed that these associations were still obtained after controlling for caregiver age and sex and PWD dementia severity, cognitive impairment, and neuropsychiatric behavior (emotional well-being ß = .18, p = .012; depression ß = -.15, p = .039; anxiety ß = -.15, p = .044). Future studies should determine whether interventions that increase co-experienced positive emotions among caregiver-PWD dyads help buffer caregivers from adverse mental health consequences.

